# Buffalo Medical and Surgical Association

**Published:** 1885-06

**Authors:** 


### BUFFALO MEDICAL AND SURGICAL ASSOCIATION.

*Meeting of April 7, 1885.*

The President, Dr. F. W. Bartlett, in the Chair; Dr. Frank H. Potter, Secretary.

*Hoarseness and Loss of Voice*—Dr. F. W. Hinkel read a paper with this title. (Published in full in the May number of this journal, page 435.)

In the discussion, Dr. W. W. Potter reported a case of aphonia from paralysis of the intrinsic muscles of the larynx, which he had relieved by external manipulation of the larynx, according to the method of Henry K. Oliver, of Boston, as described in the *American Journal of Medical Sciences* for April, 1870.

Dr. Van Peyma asked how old a child ought to be in order to make a laryngoscopic examination practicable.

Dr. Grosvenor asked whether tubercular phthisis could be a purely local disease.

Dr. W. S. Tremaine reported a case of paralysis of the adductor and abductor muscles on the right side, from a pen-knife wound of the throat. The puncture had wounded the eighth pair of nerves, and also some filaments of the spinal accessory. Electricity was tried without avail; the man finally recovered, however, after some months spent in out-door life.

Dr. Cronyn complimented the author of the paper for the able manner in which he had presented the subject. He believed every physician should be familiar with the use of the laryngoscope to a degree sufficient to enable him to make a correct diagnosis. Reported a case of loss of voice occurring in intermittent fever, the voice returning as the fever disappeared. This occurred several times. Each time a remedy for the fever restored the voice as well.

Dr. Hayd also called attention to the necessity of being familiar with the uses of the laryngoscope. If diseases of the throat can be cured at all, it can be done with much more certainty in their early stages. A chronic laryngitis, for instance, is very difficult to cure. Tubercular laryngitis is often evident in the throat before the lungs show any involvement whatever, according to Schoeder, the first symptom being a peculiar pallor of the roof of mouth and head.

Dr. Frederick believed that every medical school should have a chair of laryngology, so that students could be taught the use of the laryngoscope before graduation.

Dr. Hopkins said that as the specialists exist by the consent of the general practitioners, it was no more than an act of justice for them occasionally to instruct the rest of the profession upon the advances made in the branch of medicine to which they have devoted themselves. He advised becoming familiar with the use of the laryngoscope by each one practicing upon himself by the aid of reflected light.

Dr. Hinkel said he had made a laryngoscopic examination in a child two years and a half old. He hardly considered it practicable at that age, however, for the child could seldom be controlled sufficiently. He had seen cases of tubercular disease of the larynx where no other signs of constitutional infection could be found. It was possible, however, that there was present some constitutional disturbance, but of a latent character, which had as yet showed no other sign of its presence.

*The President's Retiring Address*—Dr. Bartlett then read his retiring address, in which he reviewed the work of the year just passed, and again called the attention of the association to the advisability of dividing it into sections, as he had suggested a year ago. He thought that by this means more accurate work could be done than was accomplished under the present plan.

On motion of Dr. Cronyn, a vote of thanks was given to Dr. Bartlett for the able and courteous manner in which he had presided over the meetings of the association during the past year.

Dr. Barnes presented the association with the record of its proceedings from the year of its organization, in 1845, to the year 1857, which he had found while looking over the books of his father.

The Treasurer then made his report, showing the financial condition of the society.

A number of members had not yet paid their dues, and it was desirable they should do so that the debts of the society could be met.

The President appointed Drs. Davidson, Thornton and Daniels a committee to investigate and report upon the Treasurer's accounts.

Officers for the ensuing year were then elected, as follows: President, Dr. Charles G. Stockton; Vice-President, Dr. W. W. Potter; Treasurer, Dr. F. E. L. Brecht; Secretary, Dr. Frank H. Potter; Librarian, Dr. J. B. Samo.

The society then adjourned.

